# Engagement with Health Care Providers Affects Self- Efficacy, Self-Esteem, Medication Adherence and Quality of Life in People Living with HIV

**DOI:** 10.4172/2155-6113.1000256

**Published:** 2013-10-29

**Authors:** Wei-Ti Chen, Dean Wantland, Paula Reid, Inge B Corless, Lucille S. Eller, Scholastika Iipinge, William L Holzemer, Kathleen Nokes, Elizbeth Sefcik, Marta Rivero-Mendez, Joachim Voss, Patrice Nicholas, J. Craig Phillips, John M. Brion, Caro Dawson Rose, Carmen J Portillo, Kenn Kirksey, Kathleen M Sullivan, Mallory O Johnson, Lynda Tyer-Viola, Allison R Webel

**Affiliations:** 1 Assistant Professor,400 West Campus Dr. #22110, Orange, CT 06477, School of Nursing, Yale University, Orange, CT 06477, USA; 2 Assistant Professor, Rutgers College of Nursing Ackerson Hall 180 University Avenue, Room 330 Newark, NJ 07102, USA; 3 Assistant Professor, University of North Carolina Wilmington (UNCW) School of Nursing 601 South College Road Wilmington, North Carolina, USA; 4 Professor, Institute of Health Professions CNY 36 1st Avenue Boston, MA 02116, USA; 5 Associate Professor, Rutgers College of Nursing 101 Glen Rock Road Cedar Grove, NJ 07009, USA; 6 Senior Lecturer University of Namibia Main Campus, Mandume Ndemufayo Avenue, Windhoek Block F, Room 204, 3rd Level Namibia; 7 Dean and Professor Rutgers College of Nursing Ackerson Hall 180 University Avenue, Room 302C Newark, NJ, USA; 8 Professor Texas A&M University-Corpus Christi 6300 Ocean Dr. Island Hall, Rm 329 Corpus Christi, TX 78404, USA; 9 Professor University of Puerto Rico PO Box 365067 San Juan, PR 00936-5067, USA; 10 Associate Professor University of Washington, School of Nursing PO Box 357266 Seattle, WA 98195, USA; 11 Professor and Director, Global Health and Academic Partnerships Brigham and Women’s Hospital and MGH Institute of Health Professions 36 1st Avenue Boston, MA 02129, USA; 12 École des Sciences Infirmières, School of Nursing Faculté des Sciences de la Santé, Faculty of Health Sciences Université d’Ottawa, University of Ottawa 451 chemin Smyth Road Ottawa, Ontario, CANADA; 13 Associate Professor UCSF School of Nursing Dept. of Community Health Systems San Francisco, CA, USA; 14 Professor and Chair UCSF, School of Nursing, 2 Koret Way San Francisco, CA 94143, USA; 15 Director, Nursing Strategic Initiatives Lyndon B. Johnson Hospital, Harris Health System 5656 Kelley Street Houston, TX, USA; 16 Associate Professor University of Hawaii School of Nursing McCarthy Mall, Webster 439 Honolulu, HI 96822, USA; 17 Associate Professor UCSF 50 Beale Street, Suite 1300 San Francisco, CA 94105, USA; 18 Assistant Professor MGH Institute of Health Professions 3047 Bonnebridge Way Houston, TX 77082, USA; 19 Instructor Case Western Reserve University School of Nursing Cleveland, OH 44106, USA; 20 Professor and Graduate Program Director, Hunter College, CUNY, Hunter Bellevue SON, 425 East 25 Street, Box 874, New York, NY 10010, USA; 21 Associate Clinical Professor, The Ohio State University College of Nursing 1585 Neil Ave. #344 Columbus, Ohio 43201, USA

**Keywords:** Adherence, Engagement, Healthcare providers, HIV, Self-efficacy, Self-esteem, Quality of life

## Abstract

The engagement of patients with their health care providers (HCP) improves patients’ quality of life (QOL), adherence to antiretroviral therapy, and life satisfaction. Engagement with HCP includes access to HCP as needed, information sharing, involvement of client in decision making and self-care activities, respect and support of the HCP for the client’s choices, and management of client concerns. This study compares country-level differences in patients’ engagement with HCP and assesses statistical associations relative to adherence rates, self-efficacy, self-esteem, QOL, and symptom self-reporting by people living with HIV (PLHIV). A convenience sample of 2,182 PLHIV was enrolled in the United States, Canada, Puerto Rico, Namibia, and China. Cross-sectional data were collected between September 2009 and January 2011. Inclusion criteria were being at least 18 years of age, diagnosed with HIV, able to provide informed consent, and able to communicate in the local language with site researchers. In the HCP scale, a low score indicated greater provider engagement. Country comparisons showed that PLHIV in Namibia had the most HCP engagement (OR 2.80, p < 0.001) and that PLHIV in China had the least engagement (OR −7.03, p < 0.0001) compared to the PLHIV in the Western countries. Individuals having better HCP engagement showed better self-efficacy for adherence (t = −5.22, p < 0.0001), missed fewer medication doses (t = 1.92, p ≤ 0.05), had lower self-esteem ratings (t = 2.67, p < 0.01), fewer self-reported symptoms (t = 3.25, p < 0.0001), and better overall QOL physical condition (t = −3.39, p < 0.001). This study suggests that promoting engagement with the HCP is necessary to facilitate skills that help PLHIV manage their HIV. To improve ART adherence, HCPs should work on strategies to enhance self-efficacy and self-esteem, therefore, exhibiting fewer HIV-related symptoms and missing less medication doses to achieve better QOL.

## Introduction

HIV is a chronic disease and quality of life (QOL) is an individual’s satisfaction or happiness with life in domains he or she considers important, becoming an important issue for people living with HIV (PLHIV) [1–4]. Challenges that might influence QOL include disease progression, medication side effects, stigma, disclosure, anxiety, and depression [5–9]. The length of time with HIV is also positively associated with QOL [10,11]. With these challenges, PLHIV usually consult their health care providers for assistance [3,12].

Health care providers (HCPs) play an important role from HIV diagnosis to treatment [13–15]. Teixeira, Gordon, Camhi & *Bakken* reported that HIV-positive individuals believe that HCPs need to fully understand the patient’s current situation, including physical and psychological status, to enhance clinician-patient engagement and make HCPs more supportive of patients [16]. Most of the PLHIV study participants (84%) believed that trusting their primary health care provider (HCP) is very important in the clinician-patient relationship; this trust can encourage PLHIV to share personal health information with their HCP [16]. Engagement with HCP is defined as client access to HCP as needed, information sharing, client involvement in decision making and self-care activities, HCPs respect and support of client choices, and management of client concerns [17].

Research has shown that senior HIV clinicians provide care to improve medical outcomes compared to less-experienced providers [18], which affects the QOL of HIV-positive patients. In Brazil, HIV-positive women receiving care from providers with a preventative care perspective and trained in reproductive health report a better QOL compared to HIV-positive women who went to providers lacking reproductive health training and counseling skills [19]. Similarly, other studies conducted regarding chronic diseases such as hypertension and diabetes indicated that patient-provider communications, including the quality of information exchange and verbal understanding from both sides, profoundly impact patients’ health outcome and satisfaction [20].

Adherence to antiretroviral therapy (ART) is an essential task for HIV-positive populations; patients who are adherent can avoid medication resistance, slow down the progression of HIV disease and reduce the occurrence of opportunistic infections and even death [21,22]. Studies have shown that engaged HCP relationships with HIV positive individuals enhance patients’ QOL that in turn prompts them to adhere to ART, resulting in improved patient life satisfaction compared with those relationships that are not engaged [17,23, 24].

Self-efficacy the belief in one’s own ability to succeed in specific situations is an important determinant for disease management [25,26]. Adherence self-efficacy in PLHIV is the confidence held by an individual in her or his ability to follow treatment recommendations, including specific HIV care such as initiating and adhering to ART [27]. Studies have shown that adherence self-efficacy is a key attribute in maintaining optimal medication adherence [28–30]. Perceived self-efficacy among PLHIV can sustain good ART adherence and self-care behavior with education from the HCP and other support systems if there are any challenges. Patients’ with higher self-efficacy and a greater belief in their ability to master tasks are more likely to perform well by accomplishing tasks, goals, and meeting challenges [28]. Self-efficacy and self-esteem have been shown to have a high correlation in many HIV-related research studies [31–33].

Self-esteem is an individual’s set of thoughts and feelings about his or her own worth and importance [34]. A recent report has shown a strong relationship between self-efficacy and self-esteem for HIV prevention among Hispanic women [33]. Women who reported lower self-esteem felt discouraged from seeking help, they felt vulnerable, and were not able to complete their daily chores [33].

HIV is a disease that profoundly influences the daily lives of PLHIV, therefore, QOL has been discussed from the onset of the HIV epidemic. Physiological and psychosocial aspects of HIV progression and the side effects of ART can be related to QOL among HIV-infected populations [35–37]. With an engaged HCP relationship and good ART adherence, we assumed, PLHIV should have enhanced QOL. However, we could not find any reports related to these variables in international populations, we compare PLHIV among Western countries, China and Namibia. The purpose of our multi-site international study was to examine the relationship of ART adherence, adherence self-efficacy, self-esteem, and health-related QOL with patients’ engagement with their HIV HCP. Specifically, we investigated whether regulatory factors adherence self-efficacy, self-esteem, and medication adherence were associated with QOL (measured by participants’ capability in participating in activities), health outcomes, and engagement with HCP. We also looked at whether this differs by international site.

## Methods

### Research design

This study is a part of a multi-national and multi-site cross-sectional study that examined QOL, engagement with HCP, and the strength of association among other variables such as adherence self-efficacy, self-esteem, and ART adherence.

#### Sample

A convenience sample of 2,182 PLHIV was enrolled from HIV clinics and service organizations globally. Participants were recruited from 15 sites throughout the United States (Boston, Massachusetts, 2 sites; Chicago, Illinois; Cleveland, Ohio; Corpus Christi, Texas; Durham, North Carolina; Arlington, Texas; Newark, New Jersey; New York City, New York; San Francisco, California, 3 sites; Seattle, Washington, 2 sites; and Wilmington, North Carolina). Five other countries/territories had one site each (Canada, Puerto Rico, Namibia, China, and Thailand). Thailand was the only site that used partial questionnaires; therefore, their data were not included in our calculation of sites. Cross-sectional data were collected between September 2009 and January 2011. Inclusion criteria in this project were being at least 18 years of age, diagnosed with HIV, able to provide informed consent and able to communicate in the local language with site researchers.

### Protection of human subjects

Each study site obtained individual institutional review board (IRB) approval before the study was conducted. Informed consent was secured from each study participant before enrollment in the study. The University of California at San Francisco (UCSF) functioned as the primary site for merging all the study data. UCSF IRB approval also included agreement for data sharing with all 20 sites and for keeping all data in the central data warehouse at the UCSF site. Code numbers were assigned and used to protect the confidentiality of all research participants.

### Translation

At four of the participating sites, the instruments were translated from English to the local language(s): Shanghai, China (Chinese), Bangkok, Thailand (Thai), San Juan, Puerto Rico (Spanish), and Windhoek, Namibia (Afrikaans and Oshiwambo). Each site followed established translation procedures. In Shanghai, bilingual investigators translated all of the agreed-upon instruments into Mandarin and back-translated into English to establish conceptual equivalence. In Bangkok, the instruments were translated into Thai by three translators. This translation was validated by three researchers and reviewed by two experts. In San Juan, one doctoral prepared translator translated the instruments from English to Spanish. An independent, bilingual translator translated that version of the instruments back to English; two additional experts reviewed both versions for conceptual and semantic equivalence. Finally, reliability of the translated instruments was assessed on 20 participants. In Windhoek, bilingual investigators translated the instruments into both Afrikaans and Oshiwambo, and back-translated into English to establish conceptual equivalence.

### Instrumentation

#### Demographic questionnaire

Our demographic survey included 20 items asking age, gender, ethnicity, education, adequacy of income, current CD4 count, viral load, length of HIV diagnosis, other health conditions, and HIV disease information with possible HIV transmission route.

#### Engagement with Health Care Providers (HCP)

The HCP scale is a 13-item scale in which clients rate the nature of their interactions with their health care providers on a four-point scale (1 = always true and 4 = never); a low score indicated greater provider engagement. The scale was submitted to principal components factor analysis with Varimax rotation. A one-factor solution emerged with an Eigen value of 8.6 and explained 66.5% of the variance. Cronbach’s alpha reliability estimate was 0.96 [17].

#### HIV Treatment Adherence Self-Efficacy Scale (HIV-ASES)

Adherence self-efficacy confidence in one’s ability to comply with a treatment plan has been consistently linked to adherence over time. The HIV-ASES scale, developed at UCSF, assesses patient confidence to carry out health-related behaviors (asking physician questions, keeping appointments, adhering to medication regimen) (Cronbach’s alpha = 0.92). This measure includes two subscales (Persistence and Integration), has been found to be associated with adherence to ART in our previous research publication [38].

#### Rosenberg self-esteem scale

We used this scale to measure self-esteem. It consists of 10 statements related to overall feelings of self-worth or self-acceptance. The items are answered on a four-point scale [34]. There is one total score, with a total range from 10 (low self-esteem) to 40 (high self-esteem). The observed Cronbach’s alpha reliability coefficient for the total score was 0.59.

#### HIV medication adherence

The 16 questions consisted of the following instruments: (a) a list of current ARV medications and questions to record the patients’ history of taking or stopping antiretroviral medications. This 5-item instrument recorded their current ARV medications and reasons for stopping ARV medications in the past. (b) AIDS Clinical Trial Group (ACTG) medications, including a 9-item survey measuring reasons for missed medications. Items are rated on a 4-point Likert scale (1 = never, 2 = rarely, 3 = sometimes, and 4 = often). The higher the score means less adherence to their ART medications [14,39]; (c) 3-day recall: This one-item visual analog scale, based on 30-day adherence assessment [40], accesses 3-day adherence, reporting separately for each drug along a continuum of “none of my doses” to “every one of my doses.” (d) 30-day recall: This one-item visual analog scale [40] assesses 30-day adherence, reporting separately for each drug, along a continuum of “none of my doses” to “every one of my doses.” This scale has been shown to correlate with other adherence measures such as MEMS caps (Medication Event Monitoring System, Aprex Corp., Fremont, Calif.). We used an ACTG subscale to calculate the participants’ adherence.

#### Quality of life

QOL was measured with Veterans Short Form (VSF)-12 Quality of Life. We used the physical domains as the variable to measure whether the study participants were capable of participating in activities as they wanted to [41–43]. VSF-12 is a 12-item health questionnaire developed from the U.S. Veterans Health Study spanning physical to psychological domains. The VSF-12 questionnaires have been administered nationally by the Veterans Administration (VA) where there have been more than 2.5 million administrations since 1996. The VSF-12 has undergone extensive testing and was shown to be reliable and valid in ambulatory care patient populations [44].

### Data management

Each site was responsible for its own data collection, ensuring completeness of the survey, entering, cleaning, and securing the data. After these tasks were completed for all participants at each site, the data were de-identified and sent to the coordinating center at the UCSF School of Nursing, Department of Community Health Systems. Once received, the data were cleaned and aggregated in SPSS 19, instrument sub-factor and total scores were calculated, and the final data file was sent to each site director for analysis. The analysis plan was collaboratively developed in October 2010 in Newark, New Jersey, USA, with all the site principal investigators.

### Data analysis

Data analyses included descriptive statistics, t-tests, odds ratios, and ANOVAs. All tests were conducted using IBM SPSS 19 and Amos19 statistical packages.

## Results

The total sample (*N* = 2182) was composed of 28.6% (*n* = 625) women (at birth) and 70.3% (n = 1,534) men (at birth) and 2.4% (*n* = 52) transgendered and gender queer individuals, of whom 36.8% (*n* = 757) were African/African-American, 21.4% (*n* = 441) white, 20.2% (*n* = 415) Hispanic, 16.1% (*n* = 330) Asian/ Pacific Islander, and 3.1% (*n* = 64) Native American. Participants mean age was 45.1 years (+/−1.36) with the youngest group from China (*M* = 37.6, *SD* = 9.7) and the oldest group from Canada (*M* = 47.1, *SD* = 8.2, p < 0.001) More than a quarter (27.8%, *n* = 601) of the study participants had achieved an 11th grade education or less; 37.2% (*n* = 804) had graduated from high school and 35.1% (*n* = 759) had graduated from a two-year college or college/university with a bachelor’s, master’s or doctoral/medical/law degree. Although the sample as a whole was almost evenly divided- as those with or without children- the sample from Canada was more likely not to have children (64.8%, *n* = 57) and the sample from Namibia was more likely to have children (90.2%, *n* = 92). A more detailed demographic analysis has been reported in other papers [42,45].

Self-efficacy and self-esteem were correlated (r= 0.28, P < 0.0001). Country comparisons showed that PLHIV in Namibia have the most HCP engagement (OR 2.80, *p* < 0.001) and that PLHIV in China have the least engagement with HCP (OR −7.03, *p* < 0.0001) (Table 1). For the HCP scale, a low score indicated greater provider engagement. Individuals having better HCP engagement showed better adherence self-efficacy (*t* = −5.22, *p* < 0.0001), missed fewer medication doses (*t* = 1.92, *p* = 0.05), had *lower self-esteem* (*t* = 2.67, *p* < 0.01), reported fewer symptoms (*t* = 3.25, *p* < 0.0001), and better overall QOL physical condition (*t* = −3.39, *p* < 0.001) Table 2.

## Discussion

Sandelowski et al. reported that engagement with the provider facilitated better ART adherence [43,46–48]. The engagement between HCPs and patients can influence whether patients take medication on time and whether they miss doses [17,49], the engaged relationship is important for both the HCPs and patients. In addition, there are power struggles between HCPs and patients because patients are seeking care from their HCP [50]. Maintaining good engagements between HCPs and patients are one of the learning techniques that improve relationships and can enhance health outcomes.

In this study, physicians were the primary HCP for participants in China and Namibia. That was because the role of the nurse practitioners and physician assistants are non- existent in these countries. In the Western countries, the majority of the study participants’ HCPs were nurse practitioners and physician assistants. Therefore, it is difficult to compare whether nurse practitioners or physician assistants engaged more with PLHIV compared to physicians who provided care to the study participants in China and Namibia.

Participants in this study from Namibia had the highest engagement with their HCP, fewer self-reported missed medication doses, fewer self-reported symptoms, and better overall QOL compared to the rest of the study participants. One might be able to explain that these study participants believe they could take care of themselves by having higher self-efficacy with good self-esteem while relying on the encouragement and support from their HCPs. In addition, Namibia has a less technology driven healthcare system and patients and providers might have more face time with each other. They might also feel culturally supported. Thus, it could be that those patients that experienced physical symptoms and ART side effects reported them to their HCP sooner creating an environment for healthcare problems to be managed earlier. In contrast, Chinese study participants reported less engagement with their HCP, less adherence self-efficacy, and lower self-esteem. Living in an authoritarian environment, such as China, PLHIV might have less opportunity for negotiation with HCPs. This report supports other studies conducted in China; the stigma of HIV is so severe that HCPs sometimes discriminate against PLHIV [15,48,51–53].

Adherence to antiretroviral therapy is the key to maintaining viral suppression [54,55]. Adherence has also been associated with better quality of life, general health, daily function, social performance, mental health, and higher CD4 counts [28,56]. Factors found to be associated with non-adherence behaviors include the following HIV-related phenomena: forgetting to take pills, having insufficient HIV knowledge, having a higher viral load, using alcohol, sleeping through the dose, being away from home, having a change in routine, being busy, being too sick to take the pills, not being able to tolerate the side effects of the ART, or being depressed [57–59]. In this study, participants who engaged more with their HCP presented with higher adherence rates compared to those who had less engagement with their HCP.

In this study, adherence self-efficacy and self-esteem were correlated; it seems that these study participants were confident in keeping up with their medication adherence. They might need encouragement from their HCP to maintain the medication adherence self-efficacy. Not surprisingly, those patients’ who were engaged with their HCP missed fewer medications and had fewer self-reported symptoms. One might hypothesize that people who value engagement with their HCP remind themselves to take medications on time and have good ART adherence self-efficacy

Better engagements between HCPs and patients facilitate health information exchange on both sides. Perhaps the trust between HCPs and PLHIV leads to better engagements. Efficient communication between HCPs and patients is highly related to the best patient care decisions; if appropriate, family members should be included in the patients’ care plan. In a trustful engagement, patients can give information that makes it possible for HCPs to better understand the patients and to optimize the patients’ living conditions (for example, by modifying patient HIV management). The engagement between HCPs and patients seems to have an important impact on the major outcomes in HIV care ART adherence and QOL. Our study supports other studies showing that engagement with HCP plays a vital role in influencing the QOL of PLHIV [18,29,60]. Future studies should focus on the relative impact of these factors on disease management and HIV care, and on the extent to which enhancement of the engagement between HCPs and HIV-infected patients may advance patient QOL.

## Limitations

There are several limitations in this multi-national and multi-site cross-sectional study. First, many study participants were recruited from Western countries (Canada, United States, and Puerto Rico). Other participants were recruited from China, and Namibia. Therefore, the participants from Western countries were heavy weighted. Second, governmental policies and funding for providing care vary from country to country. In China, few HCPs are trained to provide HIV-related care [61]. Therefore, patients in China might not have been able to select the HCP in which they could engage with the best. Alternative methods to strengthen their adherence self-efficacy may have been utilized, such as peer support groups or nurses that are not considered to be their HCP. In addition, HIV ART selections were limited due to availability and funding [62]. Third, people willing to pay for imported ART out of pocket with less side effects, may have a higher rate of adherence to the ART in comparison to people who relied on the government assigned ART which might cause unpleasant side effects [62,63]. Fourth, this study did not differentiate whether participants who used ART longer have higher adherence self-efficacy and self-esteem compared to participants who used ART for shorter periods. ART naïve participants might engage better with HCP, but show less self-efficacy for adherence. Therefore, future studies should recruit both ART naïve and experienced participants to examine whether self-efficacy changed according to length of time using ART. Finally, the data analysis of this study did not include mental health (e.g., stress or anxiety), which might be a factor in changing the engagement with HCP, adherence behavior, and decreasing overall QOL.

## Conclusions

This study suggests that promoting engagement with the HCP is necessary to facilitate skills that help PLHIV-manage their HIV. To enhance ART adherence in PLHIV, HCPs should work on strategies to improve self-efficacy and self-esteem, therefore, decreasing HIV-related symptoms and missing less medication doses to achieve better QOL in the PLHIV population. Tailored interventions to strength the HCP-PLHIV engagement should be designed and tested. Future studies should evaluate whether the interventions focusing on enhancing self-efficacy and self-esteem can be translated to other cultures.

## Figures and Tables

**Table 1:** Compare Health Care Providers Engagement among Participants in China, Namibia and Western Countries (U.S., Canada, Puerto Rico).

		Mean difference	Standard Error	Significance	95% confidence Interval	
					Lower Bound	Upper Bound
Western Countries	China	−7.03	0.74	0.0001*	−8.85	−5.20
	Namibia	2.80	0.76	0.001*	0.93	4–66

* The mean difference is significant at the 0.05 level

**Table 2:** Correlations among Countries, Self Efficacy, ART Adherence, Self-Esteem, Symptoms, and Quality of life.

	β	Standard Error	Student’s *t* test	Significance	Correlations
(Constant)	7.90	2.088	3.78	0.0001	
By county/continent	6.76	0.76	8.92	0.0001*	0.25
Self efficacy Adherence scale	−0.05	0.01	−5.22	0.0001*	−0.23
ART adherence	0.10	0.05	1.92	0.05*	0.21
Rosenberg self-esteem scale	0.09	0.04	2.67	0.01*	0.21
HIV-related Sign & Symptom Check List (SSC-HIV)	0.05	0.01	3.25	0.0001*	0.24
Quality of life (Physical)	−0.07	0.02	−3.39	0.001*	−0.20

Dependent Variable- Health Care Providers Relationships

* p ≤ 0.05
